# *Dentromers*, a Family of Super Dendrimers with Specific Properties and Applications

**DOI:** 10.3390/molecules23040966

**Published:** 2018-04-20

**Authors:** Didier Astruc, Christophe Deraedt, Rodrigue Djeda, Catia Ornelas, Xiang Liu, Amalia Rapakousiou, Jaime Ruiz, Yanlan Wang, Qi Wang

**Affiliations:** 1 ISM, UMR 5255, University of Bordeaux, 33405 Talence CEDEX, France; c.deraedt@yahoo.fr (C.D.); rodrigue.djeda@gmail.com (R.D.); catiaornelas@catiaornelaslab.com (C.O.); xiang.liu@ctgu.edu.cn (X.L.); amalia.rapakousiou@imdea.org (A.R.); jaime.ruiz-aranzaes@u-bordeaux.fr (J.R.); wangyanlan419@hotmail.com (Y.W.); youling1993@126.com (Q.W.); 2 Université Jean Lorougnon Guédé, BP 150 Daloa, Ivory Coast; 3 Institute of Chemistry, University of Campinas-Unicamp, 13083-861 Campinas, SP, Brazil; 4 College of Materials and Chemical Engineering, China Three Gorges University, Yichang 443002, China; 5 Chemistry of Low-Dimensional Materials, IMDEA Nanoscience Institute, Calle Faraday, 9, 28049 Madrid, Spain; 6 Department of Chemistry and Chemical Engineering, Liaocheng University, 1, Hunan Road, Liaocheng 252059, China

**Keywords:** dendrimer, dendron, *dentromer*, sensor, catalysis, redox, ferrocene, template, micelle

## Abstract

*Dentromer*s (from dentro, δεντρο: tree in Greek), and *meros* (μεροσ, in greek: part) are introduced as a family of dendrimers constructed according to successive divergent 1 → 3 branching. The smaller *dentromers* have 27 terminal branches. With alcohol termini they were originally named arborols by Newkome, who pioneered 1 → 3 constructions of dendrimers and dendrons. Giant *dentromers* have been constructed and decorated in particular with ferrocene and other redox active groups. The synthesis, specific properties, and applications are examined in this mini review article dedicated to Don Tomalia, with an emphasis on dense peripheral packing favoring the functions of encapsulation, redox sensing, and micellar template for catalysis in water and aqueous solvents.

## 1. Introduction

Besides the seminal articles on dendrimers by Tomalia [1], Newkome [2], and Denkewalter [3] in the early 1980s, the beauty and enormous potential of dendrimers were revealed to the scientific community by the superb prospective review article published by Don Tomalia in *Angewandte Chemie* in the late 1980s [4]. Tomalia was the one who astutely coined the term dendrimer [4]. He also produced by far the most popular family of dendrimers, the polyamidoamine dendrimers known to all chemists as PAMAM dendrimers [4,5], with great potential in biomedical applications [6,7,8,9]. These dendrimers, like most of the other dendrimer families, were synthesized according to a double-branching construction called 1 → 2 branching, whereby each group is branched to two other groups, and so on, i.e., doubling the number of peripheral groups from one generation to the next [10,11,12,13,14,15,16,17,18,19,20,21,22,23,24,25,26,27,28,29,30,31,32,33,34,35,36,37,38].

In the present mini-review, we wish to highlight another dendrimer family, synthesized upon *triple branching*, therefore leading to a much larger number of tethers than double branching for the same generation number. We call this family “*dentromers*” from *dentro* (δευτρο, in Greek: tree) and *meros* (μεροσ, in Greek: part, etymologically “made of several parts,” like in *polymers*). The triple-branching dendrimer construction [39] is much less frequent than the double-branching one [40,41,42,43]. It was pioneered by Newkome in his seminal article on unimolecular micelles called arborols in 1985 [2]. The topology of triple branching is found with linkers such as a trisubstituted carbon connected to a substrate, a 1,3,5-trisubstituted arene, or gallic acid with the 3,4,5 arene hydroxy substituents and derivatives.

The first method of triple branching was extensively used by Newkome to synthesize useful dendrons [44]. For instance, derivatives of tris(hydroxymethyl)methane were shown to react cleanly with acrylonitrile to yield a tris-nitrile or with chloroacetic acid in methanol to yield tris(esters) [45,46], and nitromethane was also a starting point reacting with acrylic esters to yield tris(ester) derivatives [46,47]. Another strategy consisted of the connection of a pre-synthesized trifunctional dendron, was achieved by Newkome’s group upon the reaction of a bromo-terminated dendrimer core with a dendron bearing a focal point terminated by an ethynyl group [46,48]. Interestingly, this pre-prepared dendron grafting strategy was even applied to PAMAM dendrimers to form molecular micelles [49] and to perform calixarene core functionalization [50]. Miller and Neenan initiated an arene-cored triple-branching construction with 1,3,5-triiodobenzene when they reported their seminal convergent dendrimer construction in 1990 [11] (as Hawker and Fréchet [10]), but the convergent strategy further involved arene desymmetrization with only double branching. Finally, another family of useful dendrons is Percec dendrons derived from gallic acid, leading to the assembly of a variety of soft materials with various shapes [51].

## 2. Organoiron Arene Activation

**Scheme 1:**
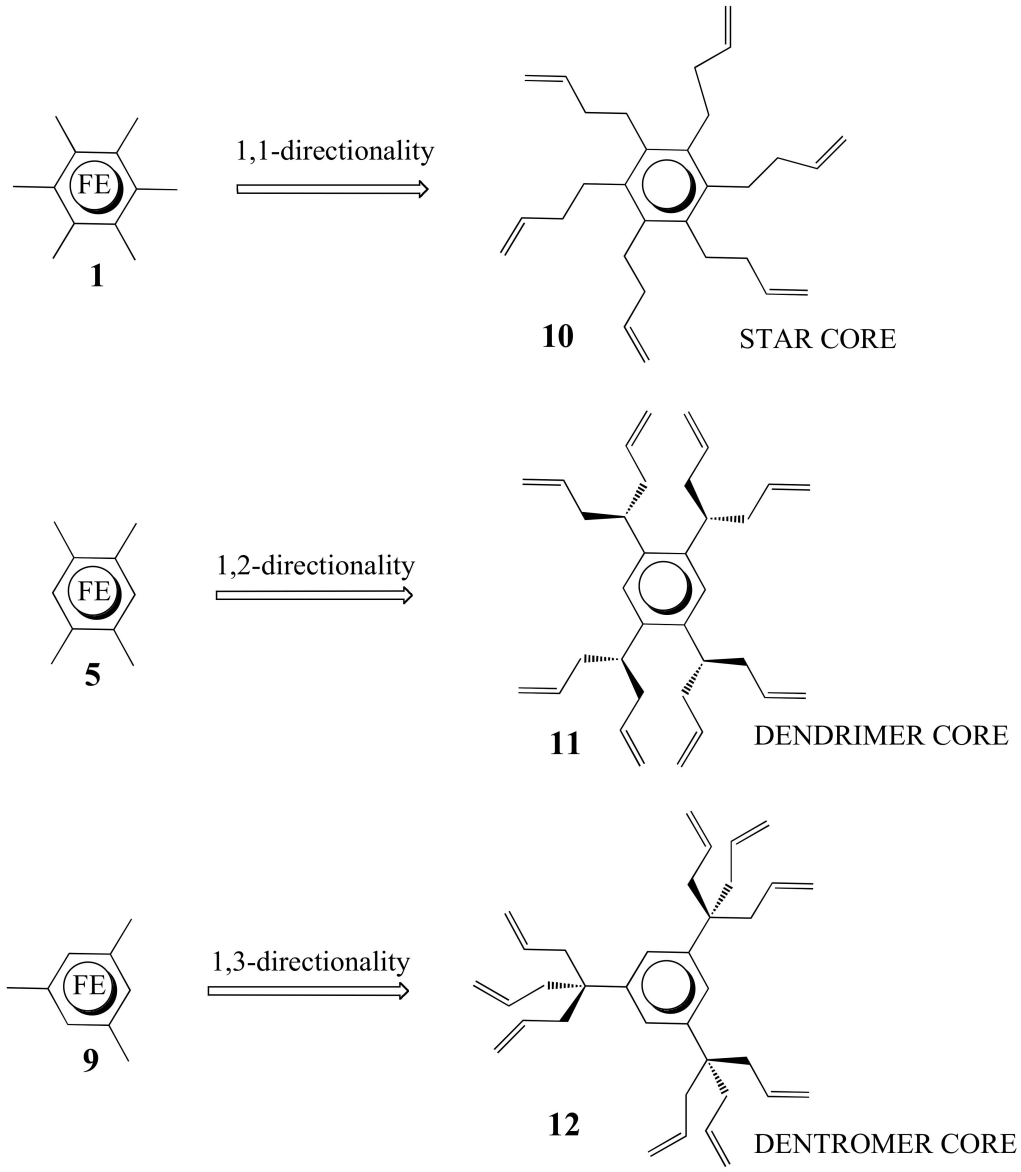
Various directionalities quantitatively obtained upon perallylation of cationic polymethylbenzene iron sandwich complexes upon reaction with KOH and allylbromide in THF under ambient conditions followed by visible-light photodecomplexation. Complex **1** = [CpFe^II^(η^6^-C_6_Me_6_)] [PF_6_]; in the scheme, FE = (η^5^-C_5_H_5_Fe^II^)^+^ with the PF_6_^−^ counter anion.

Our *dentromer* construction and other branched molecule constructions were initiated by powerful temporary organoiron activation of mono- or polymethyl arenes. The principles of activation of simple and commercial arenes for branching out are detailed as follows. The η^5^-cyclopentadienyl (Cp) iron group is indeed an excellent, simple, cheap, and non-toxic (contrary to metal carbonyls) arene activator in both the 18-electron cationic sandwich complexes [CpFe^II^(η^6^-arene)] [PF_6_] [52,53] and in the 19-electron neutral isostructural analogues [CpFe^I^(η^6^-arene)] [54,55,56]. These cationic complexes are easily synthesized on a large scale by reactions of the arenes with ferrocene [57]. In 1979, a series of six iterated sequences consisting of deprotonation/nucleophilic substitution reactions allowed us to transform the hexamethylbenzene ligand in the cationic complex [CpFe^II^(η^6^-C_6_Me_6_)] [PF_6_], **1**, into hexaethylbenzene in the complex [CpFe^II^(η^6^-C_6_Et_6_)] [PF_6_], **2**. The reactions were conducted under ambient conditions either stepwise [54] or as a one-pot reaction [58]. This was the beginning of an iteration strategy to synthesize arene-centered star molecules, including functional ones according to a 1 → 1 directionality [59]. Upon reaction of complex **1** with *t*-BuOK and allyl bromide, it was possible to selectively synthesize from complex **1** the hexabutenyl benzene complex [CpFe^II^(η^6^-C_6_(CH_2_CH_2_CH=CH_2_)_6_)] [PF_6_], **3** (see the arene ligand structure of **3** in scheme 1) in one day or, much more slowly, the dodecaallyl dendritic molecule [CpFe^II^{η^6^-C_6_[CH(CH_2_CH=CH_2_)_2_]_6_}] [PF_6_], **4** upon reaction in three weeks. This later reaction corresponds to a 1 → 2 directionality [60]. This double branching with allyl groups was much more easily obtained by utilizing the same reaction starting from the 1,2,4,5-tetramethylbenzene (durene) complex [CpFe^II^(η^6^-1,2,4,5-C_6_H_2_Me_4_)] [PF_6_], **5** [60] or from the other 18-electron complex [CpCo^III^(η^5^-C_5_Me_5_)] [PF_6_], **6** [61], providing the dendrimer cores [CpFe^II^(η^6^-1,2,4,5-C_6_H_2_{CH(CH_2_CH=CH_2_)_2_}_4_)][PF_6_], **7** (see the arene ligand structure of **7** in scheme 1) and [CpCo^III^(η^5^-C_5_{CH(CH_2_CH=CH_2_)_2_}_5_)] [PF_6_], **8** respectively. This reactivity trend shows that the branching facilities in the polymethylbenzene complexes [CpFe^II^(η^6^-C_6_H_n_Me_6-n_)] [PF_6_] at a given methyl arene substituent strongly depends on the number of methyl group neighbors of this methyl group (Scheme 1).

This rule was establishing from the beginning with methyl iodide and was completed with the mesitylene ligand of the complex [CpFe^II^(η^6^-1,3,5-C_6_H_3_Me_3_)] [PF_6_], **9** in which the methyl substituents have no neighbors [58]. In this latter case, no steric inhibition prevents the one-pot replacement of the three H atoms of each methyl groups by 3 methyl or allyl groups upon a series of nine iterative deprotonation/methylation or allylation sequences. In this case, the reactions correspond to 1 → 3 directionality. The bursting one-pot nona-allylation of the mesitylene complex is especially interesting because it is virtually quantitative and quickly provides a nonafunctional dendritic (*dentromeric*) core **9** [62]. In addition, the arene ligand is readily displaced by mesitylene upon visible photolysis to regenerate the starting complex **9**. Altogether, after photochemical decomplexation using visible light, the star core **10**, the dendrimer core **11** and the *dentromeric* core **12** are readily accessible upon temporary complexation of the precursor haxamethylbenzene, durene, and mesitylene, respectively.

**Scheme 2:**
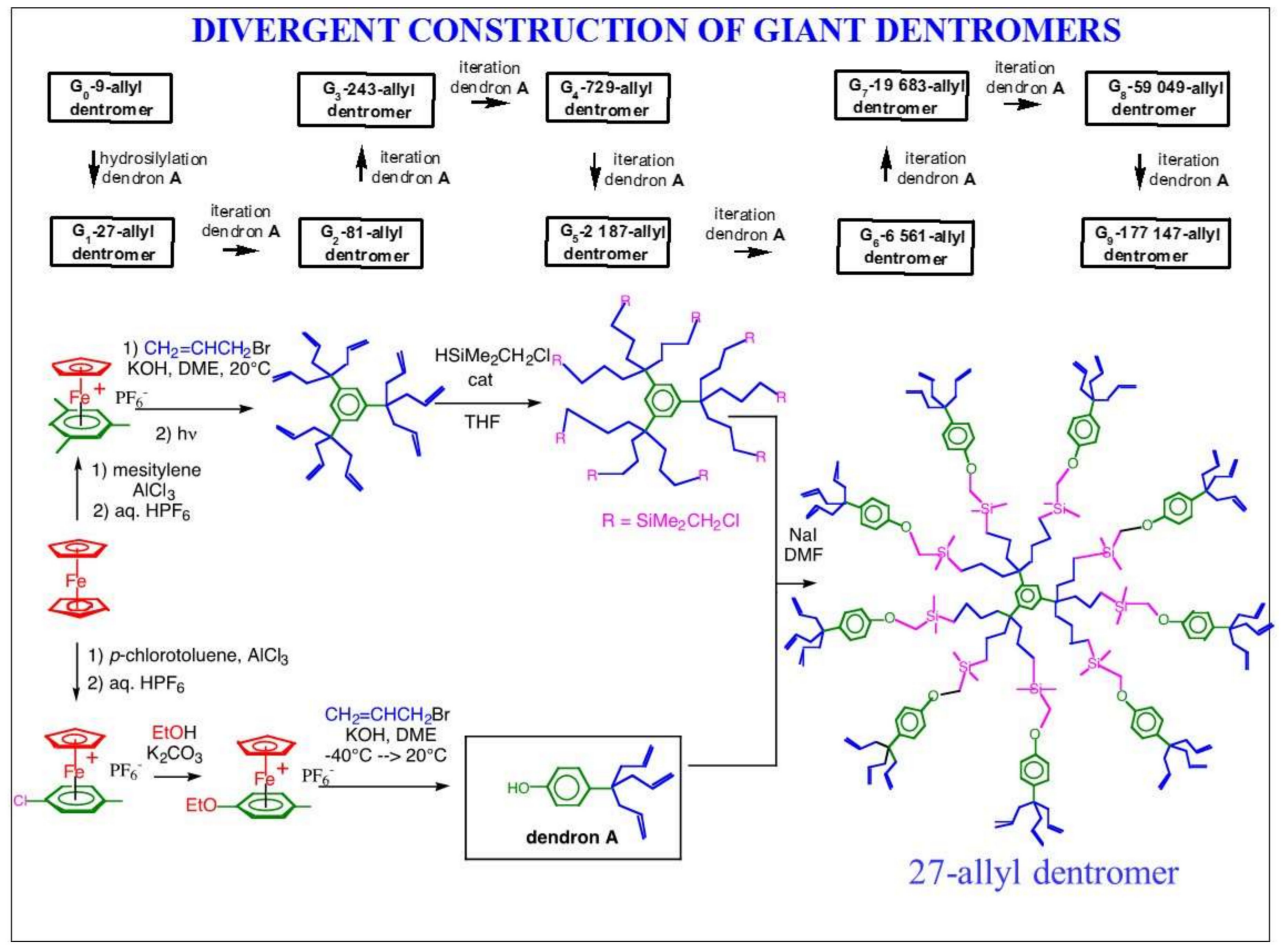
Construction of giant *dentromers* starting from ferrocene.

Another challenge was the extension of these multiple iteration sequences to the synthesis of a dendron. This was achieved starting from the *p*-chlorotoluene complex [CpFe^II^(η^6^-*p*-C_6_H_4_Me(Cl))] [PF_6_], **13**. The large-scale reaction of this complex **13** with K_2_CO_3_ in ethanol at 50 °C provided the 4-ethoxytoluene complex [CpFe^II^(η^6^-*p*-C_6_H_4_Me(OEt))] [PF_6_] that reacted with *t-*BuOK + allyl bromide to directly yield, in a one-pot reaction, the iron-free dendron *p*-HOC_6_H_4_C(CH_2_CH=CH_2_)_3_, A, in 40% yield [63], as shown in the bottom of Scheme 2. In this eight-step one-pot reaction, *t*-BuOK plays three roles: base to deprotonate the benzylic methyl groups (three times), nucleophilic cleavage of the O-C bond (assisted by CpFe^+^ activation) and finally electron-transfer reagent to transfer an electron to the iron sandwich cation, yielding the highly unstable 19-electron species [64]. Subsequent ferrocenylation upon hydrosilylation with FcSi(H)Me_2_ (Fc, ferrocenyl = η^5^-C_5_H_5_Feη^5^-C_5_H_4_) readily afforded a triferrocenyl dendron *p*-HOC_6_H_4_C(CH_2_CH_2_-CH_2_Si(Me)_2_Fc)_3_ that was convenient (with a phenol group at the focal point) for ferrocenyl-terminated *dentromer* constructions [65].

## 3. *Dentromer* Construction

**Figure 1:**
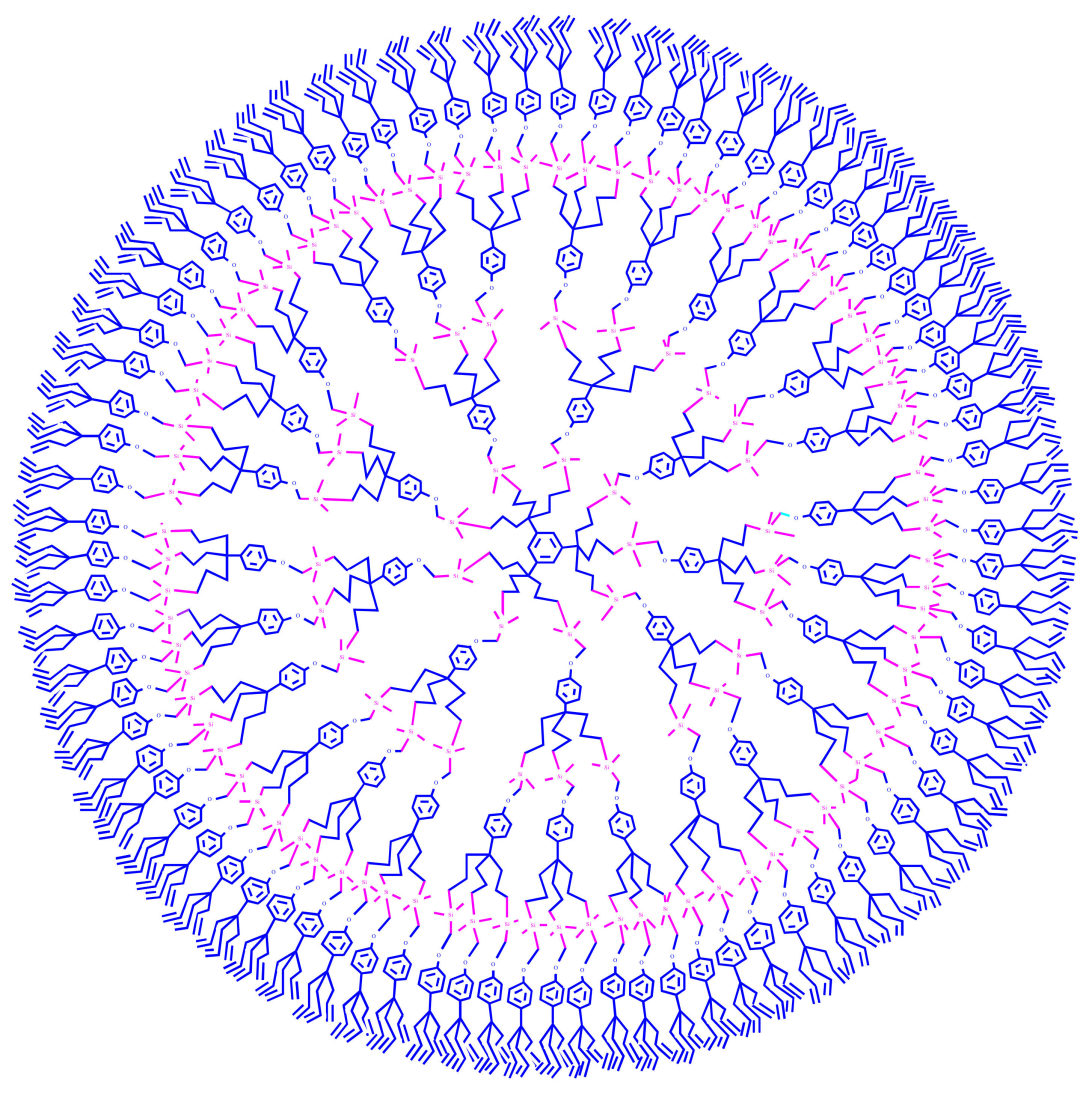
Planar representation of the 243-allyl dentromer (Scheme 2, 3rd generation).

A remarkable advantage of *dentromers* over dendrimers of the same generation is the larger number of terminal groups produced. For instance, starting from a nonabranched core (0th generation, see Scheme 2), *dentromers* reach a theoretical number of 729 terminal in only four generations, whereas 1 → 2 branching from the same core reaches only 48 terminal groups in four generations (see Figure 1 for the 3rd generation 243-allyl dentromer).

This nonaallylated core **12** was easily functionalized in many ways. For instance its hydroboration followed by oxidation by H_2_O_2_ yields a nano-ol and hydrosilylation inter alia with HSiMe_2_(CH_2_SiCl) leads to an air-robust nona-chloromethyl-terminated dendritic core in which nucleophilic substitution by NaI, NaN_3_, or sodium phenolate yields nona-iodo, nona-azido, and nonaaryloxy-terminated dendrimers, respectively, that are suitable for further Williamson or “click” reactions. At this point the introduction of additional 1 → 3 directional dendrons by appropriate coupling with the core provided clean divergent construction of *dentromers* whereby the terminal branch number is multiplied by 3 from a generation to the next one. For instance, the reaction of the nona-chloromethyl-terminated dendritic core with the phenoltriallylmethyl dendron A in the presence of sodium iodide by Williamson reaction leads to the next generation of polyallyl dendrimer with 27 allyl groups [66]. Iterative dendron branching up to the 9th generation led to a *dentromer* with a theoretical number of 177,147 branches, although the divergent construction inherently results in defects that are more numerous as the generation number increases. Nevertheless, the ^1^H, ^13^C, and ^29^Si NMR spectra, HRTEM and AFM in particular, allowed us to progressively follow the *dentromer* construction, indicating that in generation 9 the actual branch number is on the order of 10^5^ [66].

Another powerful method consisted of applying copper-catalyzed azide alkyne cycloaddition (CuAAC), i.e., “click” chemistry [67,68,69]. For instance, a reaction of the nona-azido core with the phenol dendron functionalized at the focal point with a propargyl group provides suitable 1,2,3-triazole-linked *dentromers* [70].

Thus the trifunctional dendrons may be used either to proceed from one generation to the next or to decorate *dentromers* in the same time as multiplying their number of terminal branches. Examples have been provided with dendrons that are trifunctionalized with various organometallics such as redox-active ones [71,72,73,74] or water-solubilizing ones [75]. In this later series, the Percec dendron [42,43], initially based on gallic acid, is particularly useful because the terminal hydroxyl groups are easily alkylated with triethylene glycol (TEG) bromide for water solubility.

## 4. The Applications of *Dentromers*

Starting from the nano-allylated core obtained by CpFe-induced nona-allylation of mesitylene, only one generation is needed to reach a 27-functional *dentromer* that is used as a sensor, template, and micelle, in particular for catalysis applications [76].

**Figure 2:**
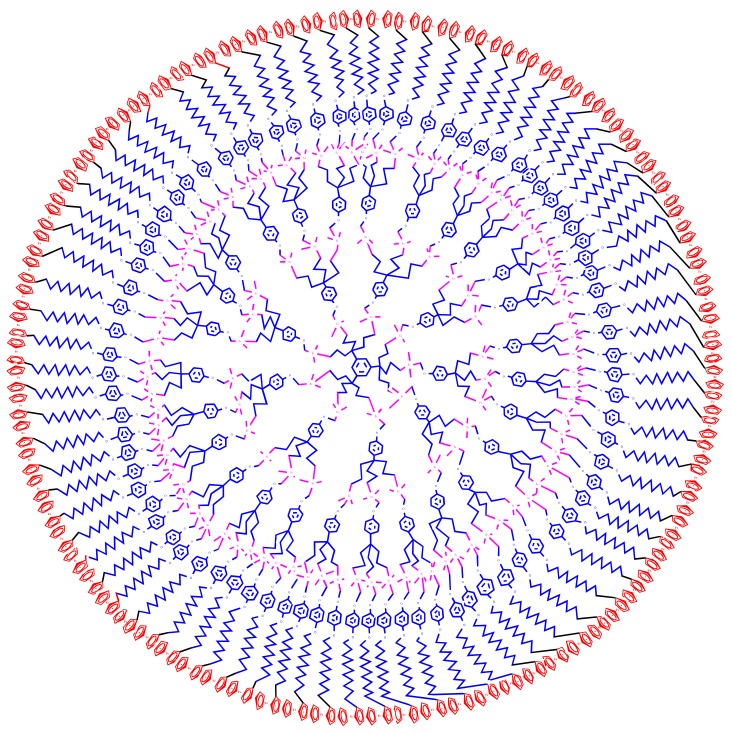
Planar representation of a 81-ferrocenyl *dentromer* (2nd generation).

Construction of ferrocenyl-terminated dendrimers (Figure 2) was achieved until the 7th generation containing a theoretical number of 19,683 ferrocenyl groups. Actually electrochemical and spectroscopic monitoring led to an estimation of approximately 15,000 terminal groups. AFM showed the steady increase of the height of the *dentromer* layer upon increasing generation numbers. The images of the 5th and 6th generations showed regularly distributed spots [72].

Cyclic voltammograms consisted of a single wave from the 0th to the 7th generation without significant irreversibility or slow electron transfer, indicating faster rotation of the *dentromer* and/or electron hopping from redox site to redox site. Oxidation of the 5th generation ferrocene *dentromer* led to a reversible size increase of nearly 50% by AFM of the *dentromer* upon oxidation, meaning that these nanosystems breathe like molecular machines upon redox cycling. The apparent equivalence of all the peripheral redox sites in cyclic voltammetry has a very important consequence for redox recognition and sensing.

**Figure 3:**
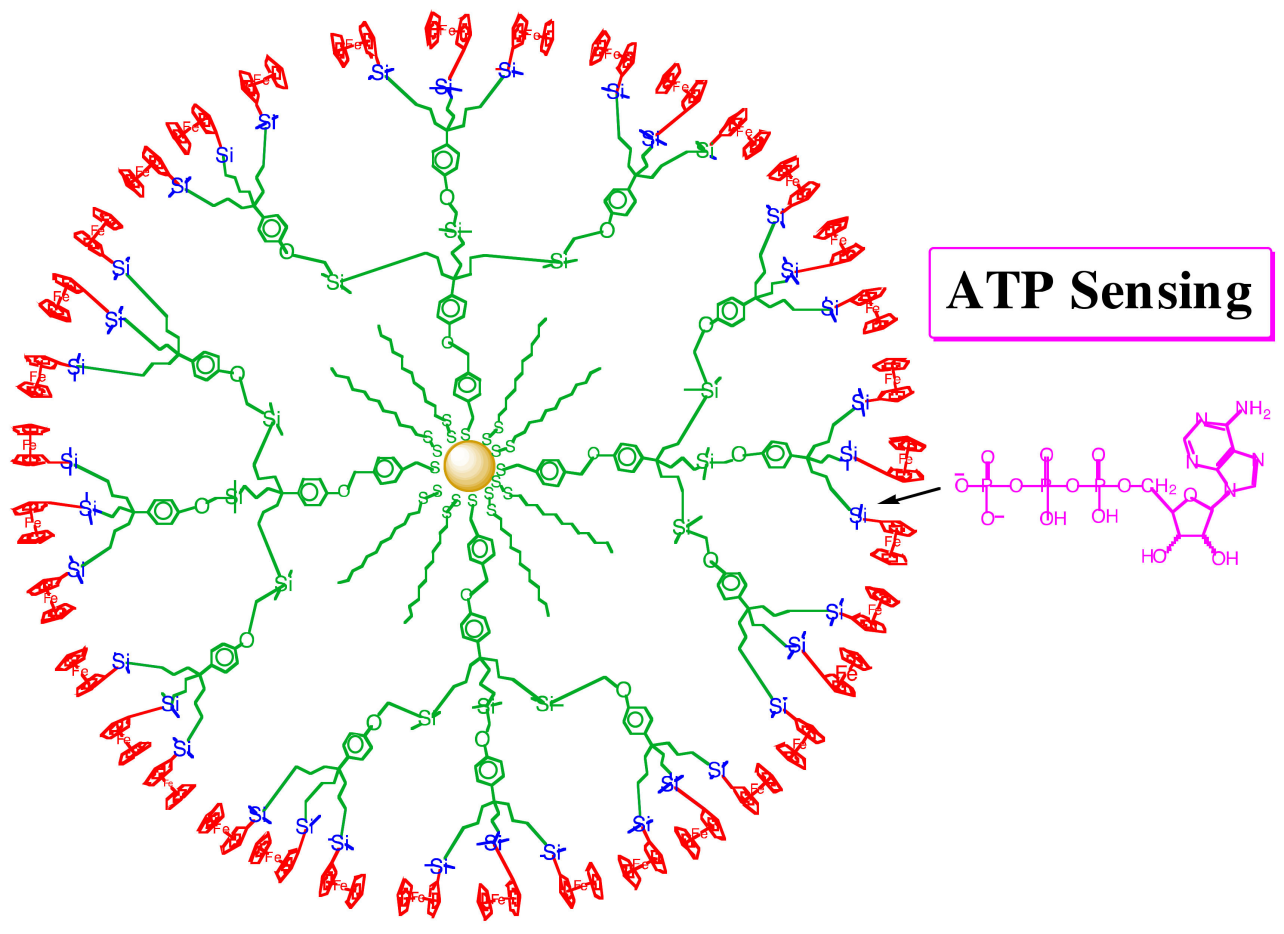
Redox ATP^2-^ sensing using a large gold NP-cored silylferrocenyl-terminated *dentromer*. The cyclovoltammetry wave of the silylferrocenyl groups interacting with ATP^2-^ is significantly shifted, allowing titration. Modified electrodes with this *dentromer* allow repetitive titrations.

Indeed, redox sensing is a useful property of *dentromers* terminated by redox-active groups such as ferrocene [77], biferrocene [74], CpFe(η^6^-arene) [77], [(η^5^-C_5_Me_5_)Fe(dppe)] [78], cobalticinium [79], or Fe_4_Cp_4_(CO)_4_ clusters [80]. Besides the ion pairing and supramolecular hydrogen bonding properties, the parameter that boosts the redox recognition of substrates is the dendritic effect involving encapsulation of substrates inside the dendrimer periphery. This crucial dendritic effect is optimized in *dentromers* because they ensure a denser packing of terminal groups with 1 → 3 branching than in looser 1 → 2 branching. This phenomenon appears particularly marked in amidoferrocenes [81,82] and click ferrocenyl dendrimers, which sense both oxo-anions such as ATP^2-^ (a DNA fragment) [83] and transition-metal cations [70,74]. The combined use of gold nanoparticles (NPs) and dendrons organized with 1 → 3 connectivity such as nanosilylferrocene dendrons with a thiol terminal group allows us to construct large ferrocenyl *dentromers* in which the weak interaction between the silicon atom bonded to ferrocene and the anionic phosphate group of ATP^2-^ also provides a good means of ATP^2-^ redox sensing (Figure 3).

Another area in which *dentromers* have an additional advantage compared with dendrimers is that of support and template in catalysis. PAMAM dendrimers have already been shown to be efficient templates for catalysis of olefin hydrogenation and cross carbon–carbon coupling reactions by dendrimer-encapsulated NPs [84,85]. “Click” *dentromers* are advantageous alternatives, however.

The first catalytic applications of “click” *dentromer*-encapsulated NPs were conducted for olefin hydrogenation and the Suzuki–Miyaura reaction [70]. These *dentromers* are terminated by triazolylferrocenes, and cyclic-voltammetry-monitored titration indicated that they coordinate, for instance, with one triazole group per Pd^II^. Reduction of Pd^II^ to Pd^0^ atoms led to Pd NP formation. The sizes of these NPs, determined by transmission electron microscopy, showed that for the 27- and 81-*dentromers* they contained a number of atoms corresponding to the same number of previously coordinated Pd^II^ cations. These numbers are 36 atoms (27 + 9) for the 27-*dentromer* and 117 atoms (81 + 27 + 9) for the 81-*dentromers*. The theoretical sizes were 1.0 and 1.6 nm for the 27- and 81-*dentromers* respectively, and the observed sizes were 1.1 ± 0.2 nm and 1.6 ± 0.3 nm, respectively, showing that the agglomerated atoms were retained inside the *dentromers* in *dentromer*-encapsulated NPs. This information was useful to understand the catalytic reaction mechanisms. Whereas, for hydrogenation, the observed TOFs were as expected, the NP size was smaller, as already known for polymer-stabilized NPs [86]; in Suzuki–Miyaura reactions, the TOF was hardly dependent on the dendrimer generation, reaching values of 5 × 10^5^. This observation led to the suggestion that a leaching mechanism was in operation. In this mechanism catalysis is probably ensured by the leaching of single atoms or small clusters containing a few atoms subsequent to aryl halide oxidative addition onto the NP surface. These active atoms are supposedly more easily caught by the mother NP as the NP catalyst is higher, which would explain the “homeopathic” catalysis [70,87]. This behavior is related to that observed by de Vries’ group for a Heck reaction conducted at 150 °C [88], but, interestingly, the Suzuki–Miyaura reactions analyzed here, unlike the Heck reactions, were carried out under ambient conditions.

**Scheme 3:**
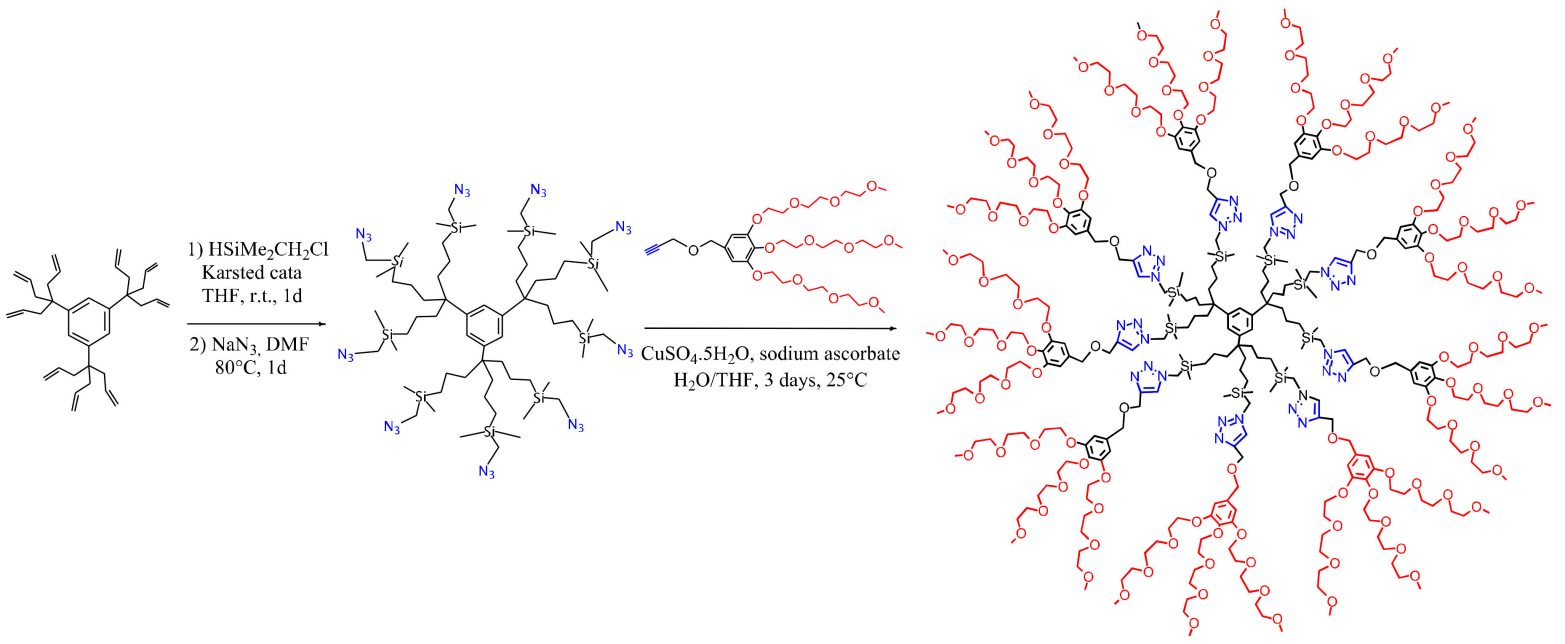
Synthesis of the standard water-soluble *dentromer* terminated by Percec-type dendrons serving as micellar template for a variety of reactions catalyzed by transition-metal complexes or NPs in water or aqueous solvents.

The small water-soluble *dentromer* containing nine Percec-type dendrons with a propargyl group at the focal point connected to the nonaazido dendritic core by CuAAC click reaction (Scheme 3) turned out to be remarkably productive and recyclable in several types of catalysis conducted in water or aqueous solvents, including olefin metathesis catalyzed by commercial Ru-benzylidene catalysts, the click reaction catalyzed by Cu^I^-tren catalyst, and several NP-catalyzed reactions [89]. Reactions were compared using either this 27-*dentromer* or the higher-generation 81-*dentromer* terminated by the same Percec dendron, and they did not result in significant dendritic effects, i.e., the TOFs did not improve upon using the 81-*dentromer* template compared to the lower-generation *dentromer*.

For instance, the use of the 27-*dentromer* allows us to decrease the amount of CuSO_4_ catalyst to ppm amounts followed by reduction to Cu^I^ by Na ascorbate and to apply this catalyst to the synthesis of triazole molecules of biomedical interest. In other examples, the same 27-*dentromer* served as a template to catalyze the CuAAC or carbon–carbon coupling reactions using ppms of Cu or Pd NP catalyst, respectively [90]. The presence of intra-*dentromer* ligands was essential in dendrimer-encapsulation NP catalysis, as demonstrated by the sizes of Au NPs obtained in 81- *dentromers* with or without inter-*dentromer* triazole ligands.

With intra-*dentromer* triazoles, the size of Au NPs formed by the addition of HAuCl_4_ followed by reduction using NaBH_4_ was approximately as expected from the number of triazole groups showing intra-*dentromer* formation, whereas without triazole groups the Au NP size obtained was much larger, indicating AuNP formation outside the dendrimer [73] Using HRTEM and HAAD STEM, it was possible to precisely locate Au NP [91] and Ag NPs [92] in nanosnakes or at the inner periphery of *dentromers*. As illustrated in Figure 1, NPs can accommodate dendrons upon binding to the core, which can also provide very efficient catalysts [93].

**Figure 4:**
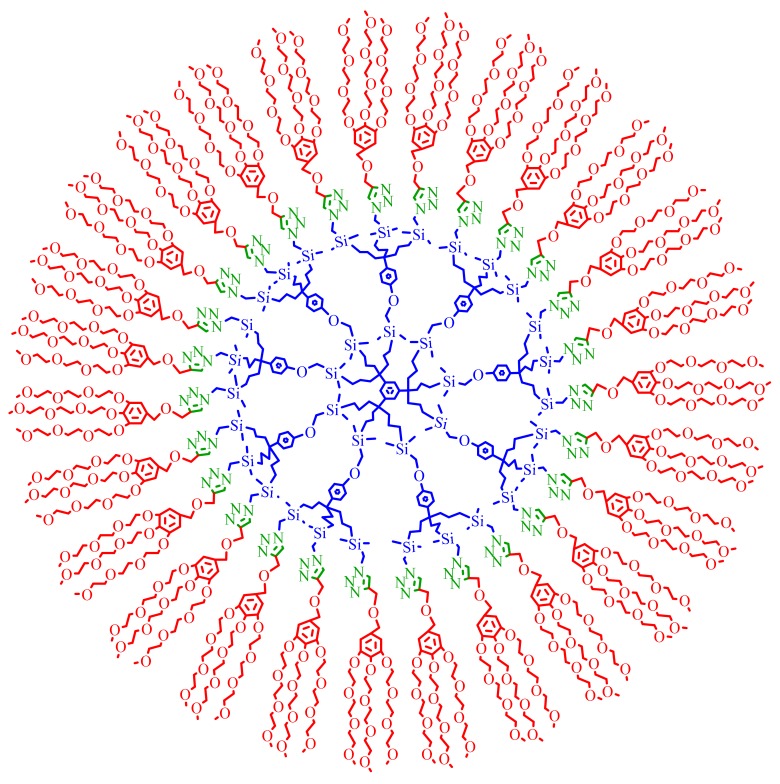
Planar representation of a 81-TEG *dentromer* (1rst generation).

Finally, the zeroth (Scheme 3) and first-generation *dentromers* (Figure 4) terminated by water-solubilizing TEG-Percec dendrons are among the best supports for catalyzing hydrogen production from ammonia-borane hydrolysis under ambient conditions, showing the potential of new applications of *dentromers* in the field of energy [94].

**Figure 5:**
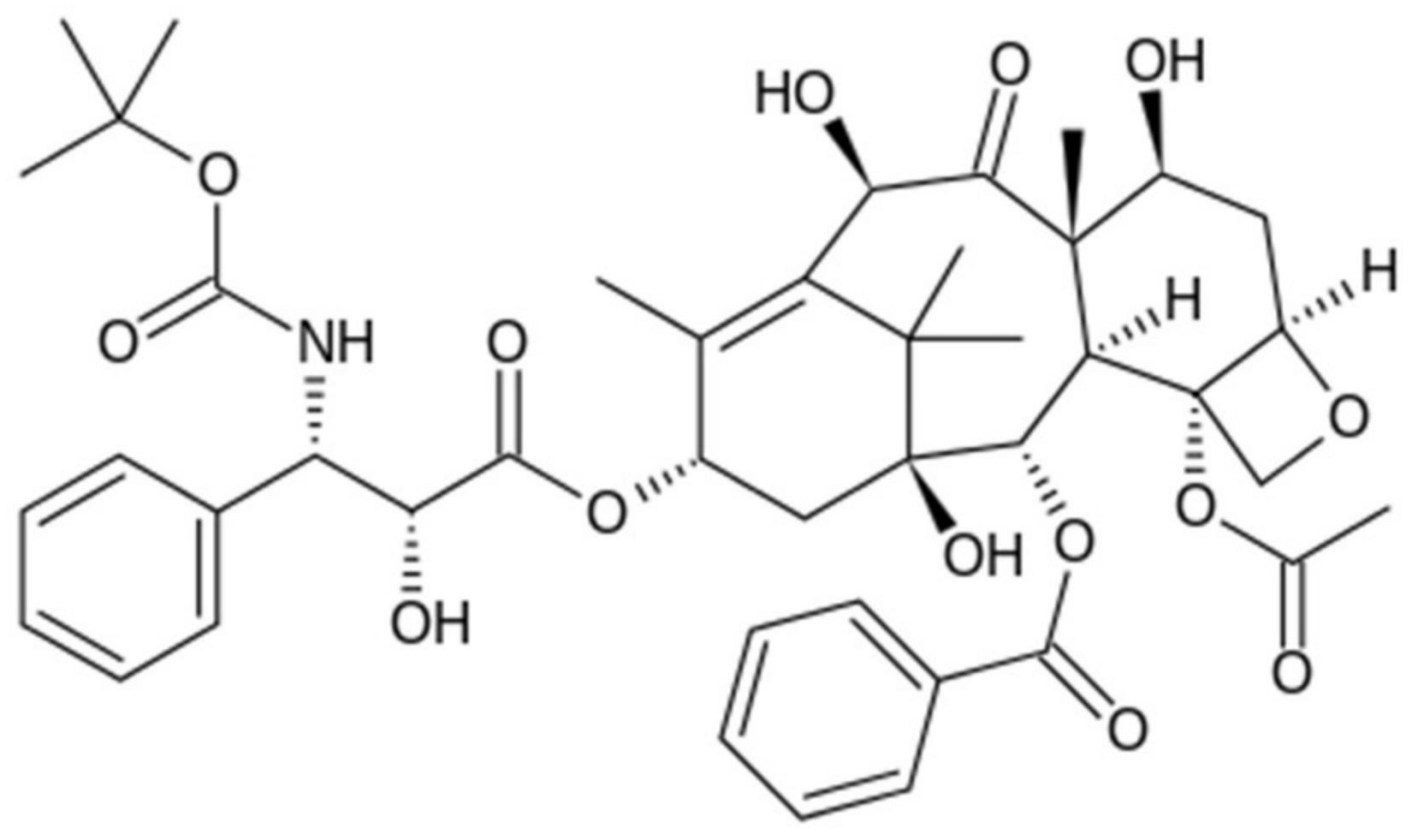
Molecular structure of the strong anti-cancer drug docetaxel.

D*entromers* have so far scarcely been applied to the biomedical field unlike the other dendrimers [6,7,8,9,32,33,34,35,36,37,38,95]. Nevertheless, gold nanoparticle-centered silylferrocenyl-terminated *dentromers* have been shown to electrochemically recognize the ATP anion, a DNA fragment [81]. In this case, *dentromers* present a significant advantage over other dendrimers, because a close dendrimer–substrate interaction is favored by triple branching, as shown by a positive dendritic effect (i.e., the fact that the increase in the generation number results in better recognition [77], unlike in catalysis [94]). *Dentromers* have also been shown to encapsulate various substrates [96,97,98,99,100], including vitamin C and other vitamins [96,97], dopamine, and acetylcholine [98,99]. It has not been demonstrated that gold-nanoparticle-cored *dentromers* would necessary present an advantage over other dendrimers in drug delivery, however. For instance, water-soluble TEG-terminated gold nanoparticles were shown by 600 MHz ^1^H NMR in D_2_O to encapsulate nine molecules of docetaxel (Figure 5). This taxane, known in its classic formulation as taxoter, is one of the most powerful and efficient anti-cancer drugs; thus, this nanodevice was designed for in vitro drug delivery [101,102].

## 5. Concluding Remarks

The original organoiron chemistry of arene activation and multiple functionalization was used in the late 1970s to propose a set of reactions leading to stars and dendritic cores. The principle of iterations has since then been the key to dendritic, dendronic, and *dentromeric* constructions. Around the turn of the century, new dendrons and giant *dentromers*, a family of dendrimers with 1 → 3 branching, were constructed based on these principles. Specific applications include the fast synthesis of *dentromers* with a large number of peripheral groups, such as ferrocene and other organometallics, leading, for instance, to molecular batteries and redox sensors. This redox behavior parallels that of the polyelectronic reduction of fullerenes [103,104], but with an electrostatic effect that is almost nil in dendrimers and *dentromers* terminated with redox groups. The fast access to small *dentromers* terminated with Percec-type dendrons also allowed many applications as micellar templates for catalysis in water or aqueous solvents. The shortcomings of *dentromers* are not apparent, although biomedical applications remain rare (vide supra); such applications might be of great interest [95].

In conclusion, the ideas and works presented in this mini-review could be developed in part thanks to the concept of dendrimers developed by Tomalia in his seminal 1990 review.

## Supplementary Materials

Supplementary File 1
