# Supplementary material for: Dentromers, a Family of Super Dendrimers with Specific Properties and Applications
Source: Molecules. 2018 Apr 20;23(4):966. doi: 10.3390/molecules23040966 (PMC6017694; doi:10.3390/molecules23040966)
Supplement: Supplementary File 1 [file molecules-23-00966-s001.pdf]

Answers to Commentaires:

The uploaded proof file is not corrected, but all the corrections are below. Thank you very much for your very efficient and fast proof preparation and, in advance, for the corrections. Below please find first all the answers to the commentaires, then the additional corrections.

Commentaire [1]: Yes

Commentaire [2]: Although the only address were the work was done by all the authors is the one that is indicated there, that is the University of Bordeaux, the present addresses of several co-authors are now different, and it is thus necessary to add a footnote either at the bottom of the first page or just before the reference list, as follows:
Present addresses:
D.A., C.D., J.R. and Q.W.: University of Bordeaux, ISM, UMR 5255, 33405 Talence Cedex;
R.D.: Université Jean Lorougnon Guédé, BP 150 Daloa , Côte d'Ivoire;
X.L.: College of Materials and Chemical Engineering, China Three Gorges University, Yichang, Hubei 443002, China;
C.O.: Institute of Chemistry, University of Campinas-Unicamp, 13083-861 Campinas, SP, Brazil;
A.R.: Chemistry of Low-Dimensional Materials, IMDEA Nanoscience Institute, Calle Faraday, 9, 28049 Madrid
Y.W.: Liaocheng University, department of chemistry and chemical engineering, 1, Hunan Road, Liaocheng, 252059 China.

Commentaire [3]: the department ISM, UMR 5255 is already indicated and nothing else need be added. No change there.

Commentaire [4]: Tel. +33 540 00 62 71

Commentaire [5]: No

Commentaire [6]: Yes

Commentaire [7]: Yes

Commentaire [8]: Yes

Commentaire [9]: OK

Commentaire [10]: by 3 methyl or allyl groups

Commentaire [11]: OK

Commentaire [12]: one number

Commentaire [13]: these abbreviations are frequent and need not be defined

Commentaire [14]: OK

Commentaire [15]: frequent abbreviation need not be defined

Commentaire [16]: OK

Commentaire [17]: confirmed

Commentaire [18]: OK

Commentaire [19]: In the reference list, exchange references [86] and [87]: ref [87] becomes ref [86] and vice-versa,
and 3rd line of page 8, replace [70,86] by [70,87]

Commentaire [20]: No need further defining (frequent abbreviation)

Commentaire [21]: OK

Commentaire [22]: replace ref 82 by: Daniel, M.-C.; Ruiz, J.; Astruc, D. Supramolecular H-bonded Assemblies of Redox-Active Metallodendrimers and Positive and Unusual Dendritic Effects on the Recognition of $H_2PO_4^-$. *J. Am. Chem. Soc.* **2003**, *125*, 1150-1151.
Replace reference [89] by Diallo, A. K.; Boisselier, E.; Liang, L.; Ruiz, J.; Astruc, D. Dendrimer-induced Molecular Catalysis in Water: the Example of Olefin Metathesis. *Chem. Eur. J.* 2010, 16, 11832 – 11835

Commentaire [23]:
Title: Macromolecular highly branched homogeneous compounds
Day, Month: 18 October

Commentaire [24]: Losada, J. Redox-active ferrocenyl dendrimers and polymers in solution and immobilized on electrode surfaces.

Commentaire [25]: the title is: A Review of Dendritic Macromolecules
Replace p3 by pp 1-67

Commentaire [26]: OK

Commentaire [27]: Yes

Commentaire [28]: 22

Additional corrections:

Page 2, section 2. Organoiron Arene Activation, Line 4: replace non-oxic by non-toxic

Page 2, section 2. Line 15: $[CpFe^{II}(\eta^6\text{-}C_6(CH_2CH_2CH{=}CH_2)_6]$ is missing a “)”

Page 2, section 2. Line 16: $[CpFe^{II}(\eta^6-C_6\{CH(CH_2CH=CH_2)_2\}_6]$ is missing a ")"

Page 2, section 2. Line 16: $[CpFe^{II}(\eta^6-1,2,4,5-C_6H_2Me_6)]$ replace Me_6 by Me_4

Page 3, caption of scheme 1, line 2: replace "+" by "and"

Page 3, last line of paragraph 1: replace "haxamethylbenzene" by "hexamethylbenzene"

Page 3, line 4 of paragraph 2: replace "+" by "and"

Page 5, line 4: replace "Na phenolate" by "sodium phenolate"

Page 5, line 9: replace "phenol dendron" by "phenoltriallylmethyl dendron A"
